# eConsultation in Plastic and Reconstructive Surgery

**Published:** 2011-11-30

**Authors:** M. J. Trovato, A. J. Scholer, E. Vallejo, G. M. Buncke, M. S. Granick

**Affiliations:** ^a^Department of Plastic Surgery, University of Texas–Southwestern, Dallas; ^b^Division of Plastic Surgery, Department of Surgery, New Jersey Medical School–UMDNJ, Newark; ^c^Buncke Clinic, San Francisco, CA

## Abstract

**Objective:** Early studies of plastic surgery patient triage using telemedicine are descriptive and deal with feasibility rather than accuracy. The inpatient study arm compares on-site wound-evaluation accuracy with remotely viewed digital images. The outpatient arm prospectively compares on-site and remote diagnosis, management, and outcomes in a busy, urban, reconstructive-surgery clinic. The concurrent 6 patient case studies illustrate significant systems improvement by using remote consultation. **Methods:** A total of 43 inpatients and 100 consecutive outpatients were evaluated by on-site and remote surgeons as performed in previous arms with digital-camera and store and forward technology. Consent was obtained from all patients participating. Agreements regarding diagnosis (skin lesion, hand injury, wound type, and scar character) and management (healing problem, emergent evaluation, antibiotics, and hospitalization) were calculated. **Results:** In the first study arm, on-site and remote agreement (46%-86% for wound description and 65%-81% for management) generally matched agreement among on-site surgeons (68%-100% and 84%-89%). Moreover, when on-site agreement was low (68% for edema), agreement between on-site and remote surgeons was also low (57%). Remote evaluation was least sensitive detecting wound drainage (46%). On-site surgeons opted for more treatment, often prescribing antibiotics and admitting the patient. The second teleconsult arm provides further evidence of accuracy, overall agreement of 32%, sensitivity 48.55%, specificity 96.92%, positive predictive value 49.26%, negative predictive value 96.83%, and *P* < .001 regarding diagnosis (skin lesion, hand injury, wound type, wound problem, and scar character). Patient transfer, postoperative monitoring, and outcomes via electronic image transfer, as well as cost-benefit analysis of this clinic-based study, are presented. **Conclusions:** eConsultation renders similar outcomes to standard, on-site examination in a selected group of plastic surgery patients. Remote evaluation may assist triage decisions, thereby decreasing emergency room throughput time and office-visit frequency, supplementing satellite facility consultation by plastic surgeons, and providing real-time postoperative assessments, thereby improving quality and reducing costs.

Telemedicine is in broad use in radiology and cardiology, where the electronic transmission and initial evaluation of radiographs and electrocardiographic tracings improve the efficiency of clinical care.^1^ The use of this technology has increased since 2002, when it was reported that 300 programs in the United States generated 250 000 consults a year in both military and civilian health care delivery systems.^2^ Plastic surgery patients frequently have conditions readily evaluated by visual inspection. Furthermore, plastic surgeons routinely photograph wounds and areas of pathology for documentation and future reference. Evaluation and triage of plastic surgery patients using telemedicine have become a topic of great interest. We have become increasingly comfortable with digital technology and recognize its value in a visually oriented clinical field of medicine. Thus far, studies have been descriptive and relatively small, and few have addressed the accuracy and concordance of surgical patient evaluation using store and forward technology. Several studies have stressed the standardization of digital photos and the use of high-quality digital imaging in evaluation of wounds and triage of injuries.

In 1998, Stoloff et al^3^ concluded that e-mail and Internet were the only cost-effective means of shipboard telemedicine. In that study, an estimated cost savings was $4400 per MEDEVAC. In 2004, Tsai et al^4^ utilized Teleconsultation by using a mobile camera phone for remote management of severe extremity wounds. They found gangrene, necrosis, erythema, and infection to be 80%, 76%, 66%, and 74%, respectively. In 2005, Hsieh et al^5^ found sensitivity and specificity of recognizing digital replantation potential, 90% and 83%, respectively. The 2005 tsunami was the first global news event where news coverage was primarily due to citizen journalists. In 2006, Katz et al^6^ used a telemanipulator slave robot to perform microvascular anastomoses. In 2006, Karamanoukian et al^7^ studied the feasibility of robotic-assisted microvascular anastomoses in plastic surgery. In 2008, Taleb et al^8^ performed a telemicrosurgery feasibility study in a rat model. Later that year, Varkey^9^ used digital photography and the Internet as a cost-effective tool in monitoring free flaps. Five reexplorations in 67 cases yielded early recognition of venous congestion and flap salvage.

Given the reliance on photography for surgical outcome evaluation and achieving reproducible and valid results in research, Galdino et al^10^ proposed guidelines for the standardization of digital photographs. In another study assessing the reliability of digital images in the evaluation of burn wounds, Jones et al^11^ used the guidelines of Galdino et al^10^ and found concordance in injury assessment between transmitted digital photos and bedside examination. Among their principal conclusions was that limitations in picture quality were a major disadvantage of telemedicine. Subsequently, investigators examined the difficulties of achieving photographic standardization in clinical settings.^12^ A series of guidelines was proposed to help physicians achieve comparable quality photos.

We seek to develop an understanding of the accuracy of remote temporal and physical digital interpretation. It is our observation that the feasibility of patient triage for most plastic and reconstructive surgery consultations is less dependent on the quality of photographs than it is on the ability to process and remotely interpret such images. Standardization in the emergency room (ER) is less practical when triaging surgical patients. Because of the scarcity of studies examining nonstandardized photography with low-quality digital imaging, we set out to determine the validity in plastic surgery e-consultation and ER triage. For the purpose of this manuscript, eConsultation refers to remote interpretation and management utilizing store and forward digital images.

## METHODS

In the initial study arm, 43 wounds in 37 inpatients were photographed with a Canon A80 camera (resolution 4.0 megapixels). On-site evaluation consisted of bedside examination by 2 physicians at the time of digital imaging and transmission. Remote evaluation consisted of 2 physicians examining digital images remotely at the time of transmission and 2 physicians examining digital images remotely at later time. A questionnaire was recorded by all physicians, and agreements regarding wound description and wound management were calculated among on-site surgeons and remote surgeons and between on-site and remote surgeons.

In the second arm, 100 patients were photographed at an urban outpatient plastic surgery clinic with a Fujifilm FinePix A330 digital camera (resolution 2.0 megapixels) without standardized photography. A medical student, untrained in medical photography, took the pictures. On-site evaluation consisted of bedside examination by a surgeon at the time of digital image capture and transmission and remote evaluation consisted of digital image consultation via store and forward technology at a later date. The remote surgeon was given no information in addition to the picture. An injury questionnaire was recorded and agreements regarding diagnosis were calculated between on-site and remote surgeons with SAS statistical software program version 9.2. A series of 5 clinical cases is presented in which patient transfer decisions, home-based postoperative monitoring, and overall outcomes were improved and costs reduced via electronic image transfer. These studies were approved by the institutional review board.

## RESULTS

Inpatient examination by on-site surgeons agreed 68% to 100% for wound description and 84% to 89% for wound management (Table 1). Remote evaluation with digital images taken by Canon A80 camera (resolution 4.0 megapixels) at transmission agreed 63% to 100% for wound description and 52% to 100% for wound management (Table 1). On-site examination and remote evaluation via store and forward technology (Canon A80 camera, resolution 4.0 megapixels) showed 46% to 86% concordance for wound description and 65% to 81% concordance for wound management. On-site and remote evaluation using Fujifilm FinePix A330 digital camera (resolution 2.0 megapixels) showed overall agreement of 32%, sensitivity 48.55%, specificity 96.92%, positive predictive value (PPV) 49.26%, negative predictive value (NPV) 96.83%, and *P* < .001, regarding diagnosis (skin lesion, hand injury, wound type, wound problem, and scar character).

## CASE SERIES

### Case 1

**Figure 1:**
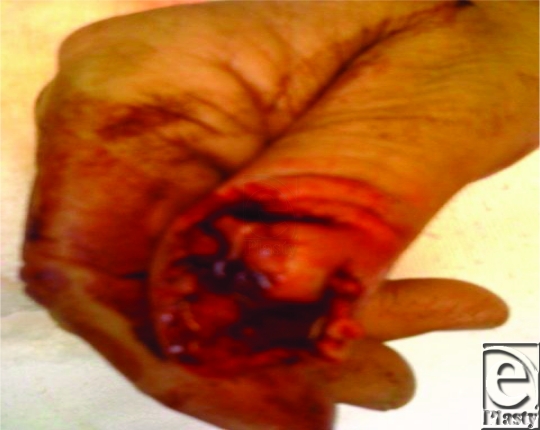


This less than optimally focused 1-megapixel iPhone image from the referring ER physician allowed assessment of injury level and exposed bone, which prompted immediate allocation of operating room resources saving approximately 1 hour of ER throughput time (Fig 1).

### Case 2

**Figure 2:**
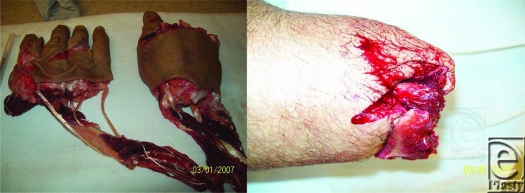


This photo series was e-mailed from a remote, referring ER, preparing to transfer the patient via fixed-wing aircraft during inclement weather for replantation. The avulsive and multilevel nature of this injury precludes replantation. The proximal stump retained sufficient soft tissue for closure without complex tissue rearrangement. Unnecessary transfer was averted on the basis of these 2 e-mailed images (Fig 2).

### Case 3

**Figure 3:**
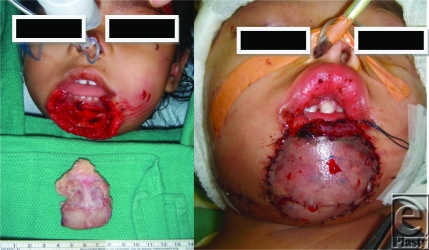


Similar images were utilized to postoperatively monitor a free tissue transfer for lower-lip reconstruction in a 2-year-old at an off-site location. Digital photographs were e-mailed twice daily, in addition to daily bedside examination by the microsurgical team (Fig 3).

### Case 4

**Figure 4:**
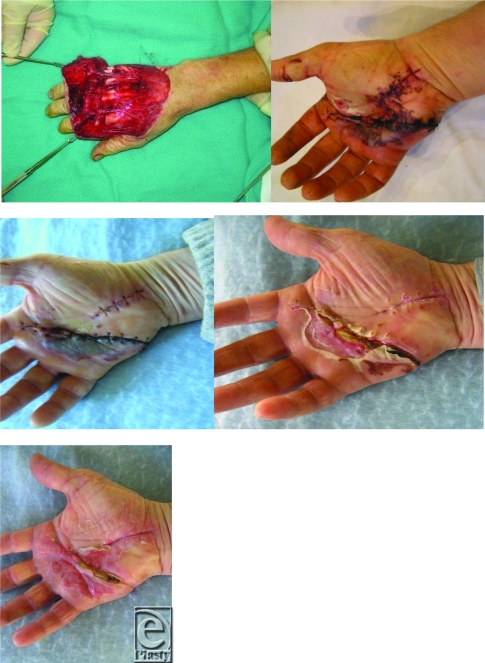


This distally based avulsion injury was monitored via twice weekly digital image series e-mailed by the patient to the surgeon. Alternatively, an injury of this type would typically mandate at least a 1-week inpatient stay for flap monitoring (Fig 4).

### Case 5

**Figure 5:**
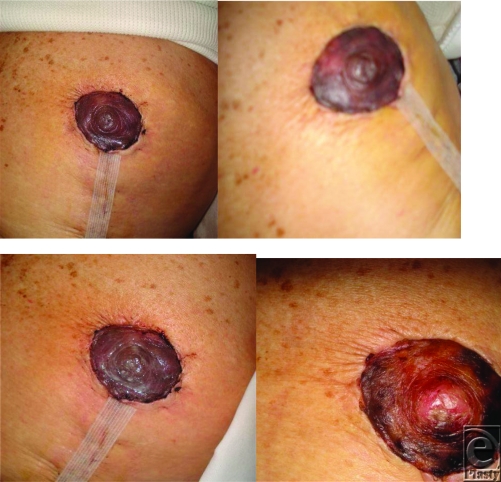


This series represents a postoperative case of questionable nipple-areolar compromise after reduction mammoplasty. On postoperative day 2, nipple compromise was deemed equivocal by on-site examination. The patient lived far from the facility and could not come in for daily evaluations, prompting a decision to closely monitor via twice daily digital photographs through e-mail. Uneventful evolution of nipple demarcation ensued over 2 weeks (Fig 5).

## DISCUSSION

The first study arm, using a Canon A80 camera (resolution 4.0 megapixels), showed 68% to 100% agreement among on-site surgeons for wound description and 84% to 89% agreement for wound management. A similar study in vascular surgery revealed comparable results among on-site surgeons (64%-85% for wound description and 63%-91% for wound management).^13^ When compared to remote evaluation at transmission, agreement among physicians was 63% to 100% for wound description and 52% to 100% for wound management. Thus, digital image evaluation of wound description correlates with bedside examination. On-site agreement was lowest for edema, erythema, and drainage, 68.4%, 73.6%, and 78.9%, respectively. Remote agreement at transmission was lowest for erythema, edema, and exposed structures, 63.1%, 73.6%, and 73.6%, respectively. Our data reveal discordance when evaluating wound description for edema, erythema, and drainage at bedside and we see the same tendency in wound evaluation by remote surgeons, using digital images at transmission. A similar pattern was documented by Wirthlin et al^13^ in 1998 for evaluation of erythema in which agreement at bedside among physicians was 64% and agreement between on-site and remote surgeons was 66%. Together, these results indicate that a decrease in agreement regarding wound description is attributed to the inherent variability in surgeon bedside examination. We compared wound description between on-site and remote evaluation at a later time using store and forward telemedicine, which showed that physicians agreed 46.6% to 86.1% (Table 1). Gangrene, necrosis, ischemia, and ecchymosis showed greatest correlation, which was consistent with the results obtained by Tsai et al,^4^ who observed 80%, 76%, 66%, and 74% agreement for gangrene, necrosis, erythema, and infection, respectively. Our data also showed a decrease in agreement for drainage evaluation (46.6%) and edema (57.6%) between on-site and remote physicians. This disagreement between on-site and remote physicians can be attributed to physician disagreement during bedside evaluation in similar areas (Table 1) and not due to store and forward technology.

A review of the trauma and burn literature reveals wound evaluation studies using high-quality digital images. Galdino et al^10^ have provided standardization guidelines with this purpose in mind.^14^^,^^15^ Our data are consistent with those in previous studies and illustrate the accuracy and reliability of wound description using store and forward technology in eConsultation with a Canon A80 camera (resolution 4.0 megapixels). For wound management, on-site physicians consistently agreed 84% to 89% (Table 1). In contrast, remote evaluation at the time of transmission varied 52% to 100% in agreement for wound management. Healing problems requiring immediate attention were recognized with 100% accuracy. On the contrary, lower concordance was achieved during remote evaluation at the time of transmission for antibiotic use and emergent evaluation (52.6%) and for hospitalization and debridement (68.4%). Furthermore, 65% to 81% agreement for wound management was achieved between on-site and remote evaluation. Remote physicians tended to be aggressive in treatment with antibiotics and increased hospital admittance and 24-hour consultation when compared with on-site physicians. Although this would seem to increase the frequency of office visits due to increased management, the reverse effect is true in practice. Triage decisions are made readily and ER throughput time is reduced, ultimately affecting health care quality and costs. We propose that containing costs and delivering effective health care can be achieved with a digital eConsult.^16^ It has been shown that using a digital photograph and the Internet allows physicians to view surgical situations and achieve increased utilization of time.^9^ eConsultation has further been shown to increase the use of same-day surgery and a decrease wait-time to physician bedside examination, thereby improving triage decisions.^17^

We hypothesized that the quality of a digital image and standardized photography used in store and forward technology do not affect diagnosis and management. On-site and remote evaluation using store and forward technology with a Fujifilm FinePix A330 digital camera (resolution 2.0 megapixels) and nonstandardization of photographs showed an overall agreement of 32% with *P* < .001 with an overall sensitivity 48.55%, specificity 96.92%, PPV 49.26%, and NPV 96.83% (Fig 3). Specificity of 96.2 and NPV 96.38 demonstrates the remote physician's ability to rule out diagnoses and correctly identify the number of people who did not have a certain condition when compared with on-site physician. Overall sensitivity 48.55 and PPV 49.26 suggest the remote physician's ability at identifying the diagnosis when compared with on-site physicians. However, the statistical analysis set the on-site physician diagnosis as “gold standard” which the remote physicians were compared. The first arm of our study showed a discord with regard to wound description among on-site physicians, which is consistent with earlier studies^4^^,^^13^ and we attribute the discord among physicians at bedside as a possible cause of suboptimal sensitivity and PPV. Moreover, our data sheet (Fig 3) was not restricted in rating criteria at the time of diagnosis. For example, if the diagnosing physicians from any of the clinical groups were originally allowed the selection of only 1 choice per category on the basis of an ordinal/stepwise level of rating instead of as many as they deemed fit, then the observation agreement would strengthen. Further studies should take into account these experimental adjustments, allowing a more precise determination of the importance of photo quality with regard to remote diagnosis.

Therefore, the original hypothesis that digital image quality and standardization of photos do not affect diagnosis and management cannot be rejected. Table 3 shows high specificity and NPV for 6 categories and 24 choice analyses. Postoperative and hand injury categories have a higher number of observations with agreement percentages similar to overall agreement and the trends in sensitivity, specificity, PPV, and NPV remain, thus strengthening the significance of our results. Therefore, the data suggest that nonstandardization of store and forward technology with low-quality digital imaging (2.0 megapixels) has similar results to a higher resolution (4.0 megapixels) when comparing on-site and remote evaluation.

ER physicians treating surgical patients who require plastic surgery consultation can use store and forward technology. Halstead et al^19^ concluded that telemedicine consult of wounds can improve triage decisions, the need to refer a patient, and help in treatment decisions. Braun et al^20^ used the mobile phone in e-dermatology wound consultation. The mobile phone remote consultation study by Tsai et al^4^ in the management of extremity wound showed a concordance in bedside examination and remote evaluation of gangrene, necrosis, erythema, and infection of 80%, 76%, 66%, and 74%, respectively. Our group set out to test the accuracy of low digital images in diagnosis and management of wounds in 100 patients. Our data indicate overall agreement: 32% with *P* < .001, overall sensitivity 48.55%, specificity 96.92%, PPV 49.26%, NPV 96.83%; category analysis: *P* < .001, agreement 26.67% to 41.18%, sensitivity 36.36% to 50%, specificity 92.22% to 96.92%, PPV 41.46% to 63.64%, NPV 90.86% to 99.49%; and 24 choice analyses: *P* value, sensitivity, specificity, NPV, and PPV (Table 3) between on-site and remote physicians using store and forward technology with a Fujifilm FinePix A330 digital camera (resolution 2.0 megapixels) regarding diagnosis (skin lesion, hand injury, wound type, wound problem, and scar character). This is comparable to on-site and remote evaluation (46.6%-86.1%) for wound description (Fig 2) via store and forward technology using a Canon A80 camera (resolution 4.0 megapixels). Thus, low-quality digital images show comparable accuracy and concordance with high-quality digital images in wound evaluation and management implicating their feasibility and practicality in remote plastic and reconstructive surgery consultation. Furthermore, digital images can improve telephone consultation by the remote plastic surgeon, ultimately assisting the ER physician in triaging patients. This is supported by the study of Pap et al,^2^ which showed improved management and treatment plans due to the addition of a photograph during a telephone call between a resident and an attending physician. This study sought to measure the accuracy of a disarmed, remote evaluator; the image was not standardized and the evaluator was given no qualifying clinical data in addition to the image. Ultimately, this study may serve to help refocus our efforts to harness the potential of telemedicine in plastic and reconstructive surgery. Our data suggest that it is less a matter of digital image focus, resolution, and bandwidth and more a matter of its timing, method of delivery, and evaluation in order to increase the efficiency of clinical decisions.

## CONCLUSIONS

Forty-three inpatients and 10 consecutive outpatients were examined on site.

Medium- and low-quality digital images were obtained without standardization and evaluated using store and forward technology. Accuracy and concordance rates were high. Variations in concordance were comparable to on-site variability. Five cases demonstrate systems improvement using eConsultation. The authors conclude that eConsultation is an accurate, cost-saving, time-saving technique in the evaluation and management of select plastic surgery patients.

## Figures and Tables

**Table 1:** Canon A80 camera (resolution 4.0 megapixels

	On-site agreement, %	Remote agreement, %	On-site/remote concordance, %
Gangrene	89.4	84.2	82.3
Necrosis	94.7	84.2	86.1
Erythema	73.6	63.1	71.4
Cellulitis/infection	89.4	89.4	76.4
Ischemia	89.4	73.6	85.2
Granulation	89.4	89.4	79.4
Ecchymosis	100	84.2	81.5
Exposed	89.4	73.6	76.4
Edema	68.4	89.4	57.6
Drainage	78.9	100	46.6
Healing	84.2	100	81.2
24-h medical doctor	89.4	52.6	70.5
Hospitalization	84.2	68.4	75
Intravenous antibiotics	84.2	52.6	65.6
Debridement	84.2	68.4	75

**Table 2:** Second-arm questionnaire

Skin lesion	Acute wound	Chronic wound
Benign	Early	Stage I
Malignant	Delayed	Stage II
Undetermined	Cellulitic	Stage III
Infected	Exposed structure	Stage IV
Postoperative	Hand injury	Scar
Wound problem	Laceration	Burn
Infected	Bony injury	Keloid/hypertrophic
Uneventful	Suspected tendon/nerve/vascular	Unfavorable
Suture removal	Late effect/deformity	Normal

**Table 3:** Second-arm negative predictive value results (P < .001)*

Skin lesion	Acute wound	Chronic wound
Benign: 96.74%	Early: 96.94%	Stage I: NA
Malignant: 100%	Delayed: 98.89%	Stage II: NA
Undetermined: 98.95%	Cellulitic: NA	Stage III: 96.94%
Infected: NA	Exposed structure: NA	Stage IV: 98.98%
Postoperative	Hand injury	Scar
Wound problem: 88.04%	Laceration: 95.6%	Burn: 100%
Infected: NA	Bony injury: 88%	Keloid/hypertrophic: 100%
Uneventful: 90.79%	Suspected tendon/nerve/vascular: 92.71%	Unfavorable: 97.6%
Suture removal: 94.74%	Late effect/deformity: 91.67%	Normal: NA

* NA indicates not applicable.

## References

[1] Dhruva VN, Abdelhadi SI, Anis A (2007). ST-segment analysis using wireless technology in acute myocardial infarction (STAT-MI) trial. J Am Coll Cardiol.

[2] Pap S, Lach E, Upton J (2002). Telemedicine in plastic surgery: E-consult the attending surgeon. Plast Reconstr Surg.

[3] Stoloff PH, Garcia FE, Thomason JE, Shia DS (1998). A cost-effectiveness analysis of shipboard telemedicine. Telemed J.

[4] Tsai HH, Pong YP, Liang CC, Hsieh CH (2004). Teleconsultation by using the mobile camera phone for remote management of the extremity wound: a pilot study. Ann Plast Surg.

[5] Hsieh CH, Jeng SF, Chen CY (2005). Teleconsultation with the mobile camera-phone in remote evaluation of replantation potential. J Trauma.

[6] Katz RD, Taylor JA, Rosson GD, Brown PR, Singh NK (2006). Robotics in plastic and reconstructive surgery: use of a telemanipulator slave robot to perform microvascular anastomoses. J Reconstr Microsurg.

[7] Karamanoukian RL, Finley DS, Evans GR, Karamanoukian HL (2006). Feasibility of robotic-assisted microvascular anastomoses in plastic surgery. J Reconstr Microsurg.

[8] Taleb C, Nectoux E, Liverneaux P (2008). Telemicrosurgery: a feasibility study in a rat model. Chir Main.

[9] Varkey P (2008). A picture speaks a thousand words: the use of digital photography and the Internet as a cost-effective tool in monitoring free flaps. Ann Plast Surg.

[10] Galdino GM, Vogel JE, Vander Kolk CA (2001). Standardizing digital photography: it's not all in the eye of the beholder. Plast Reconstr Surg.

[11] Jones OC, Wilson DI, Andrews S (2004). The reliability of digital images when used to assess burn wounds. J Telemed Telecare.

[12] Persichetti P, Pierfranco S, Langella M, Marangi GF, Carusi C (2007). Digital photography in plastic surgery: how to achieve reasonable standardization outside a photographic studio. Aesth Plast Surg.

[13] Wirthlin D, Buradagunta S, Edwards R (1998). Telemedicine in vascular surgery: feasibility of digital imaging for remote management of wounds. J Vasc Surg.

[14] Murphy RX, Bain MA, Wasser TE, Wilson E, Okunski WJ (2006). The reliability of digital imaging in the remote assessment of wounds: defining a standard. Ann Plast Surg.

[15] Jones SM, Milroy C, Pickford MA (2004). Telemedicine in acute plastic surgical trauma and burns. Ann R Coll Surg Engl.

[16] Trovato M, Scholer A, Lombardi J, Vasquez S, Granick M Telemedicine consultation in plastic and reconstructive surgery: a prospective analysis of one-hundred clinical cases.

[17] Trovato M, Scholer A, Lombardi J, Granick M eConsultation in plastic and reconstructive surgery: a prospective analysis of one-hundred consecutive clinical cases.

[18] Wallace DL, Jones SM, Milroy C, Pickford MA (2008). Telemedicine for acute plastic surgical trauma and burns. J Plast Reconstr Aesthet Surg.

[19] Halstead LS, Dang T, Convit RJ, Rosen MJ, Woods S (2003). Teleassessment compared with live assessment of pressure ulcers in a wound clinic: a pilot study. Adv Skin Wound Care.

[20] Braun RP, Vecchiett JL, Thomas L (2005). Telemedical wound care using a new generation of mobile telephones. Arch Dermatol.

